# A Treatise on Nervous Diseases

**Published:** 1885-11

**Authors:** 


A TREATISE ON NERVOUS DISEASES, THEIR SYMPTOMS AND TREATMENT. A text-book for students and practitioners. By Samuel G. Webber, M. D., Clinical Instructor in Nervous Diseases Howard Medical School, visiting Physician for diseases of the nervous system at the Boston City Hospital, member of the Massachusetts Medical Society, member of the American Nurological Association, etc., etc. New York: D. Appleton & Company, 1, 3 and 5 Bond street. 1885.

The author tells us that this book is not written for specialists Many will probably find that no department or division is so fully treated as they may sometimes wish. He trusts that students and general practitioners who have little time to read will find what they most need for diagnosis and treatment of the cases occurring in practice.

The paper and printing, with large, clear type, is a fine specimen of the good taste and superior workmanship of this establishment.

